# Stigma, depression, and quality of life among people with pulmonary tuberculosis diagnosed through active and passive case finding in Nepal: a prospective cohort study

**DOI:** 10.1186/s44263-024-00049-2

**Published:** 2024-03-24

**Authors:** Kritika Dixit, Bhola Rai, Tara Prasad Aryal, Noemia Teixeira de Siqueira-Filha, Raghu Dhital, Manoj Kumar Sah, Ram Narayan Pandit, Govinda Majhi, Puskar Raj Paudel, Jens W. Levy, Job van Rest, Suman Chandra Gurung, Gokul Mishra, Knut Lönnroth, Stephen Bertel Squire, Kristi Sidney Annerstedt, Laura Bonnett, Ahmad Fuady, Maxine Caws, Tom Wingfield

**Affiliations:** 1 Birat Nepal Medical Trust, Kathmandu, Nepal; 2 Department of Global Public Health, WHO Collaborating Centre On TB and Social Medicine, Karolinska Institutet, Stockholm, Sweden; 3 Department of Health Sciences, University of York, York, UK; 4 KNCV Tuberculosis Foundation, The Hague, Netherlands; 5 Centre for Tuberculosis Research, Departments of International Public Health and Clinical Sciences, Liverpool School of Tropical Medicine, Liverpool, L3 5QA UK; 6 Tropical and Infectious Disease Unit, Liverpool University Hospitals NHS Foundation Trust, Liverpool, UK; 7 Institute of Population Health, University of Liverpool, Liverpool, UK; 8 Faculty of Medicine, Universitas Indonesia, Jakarta, Indonesia

**Keywords:** Tuberculosis, Stigma, Depression, Quality of life, Active case finding, Passive case finding, Nepal

## Abstract

**Background:**

The psychosocial consequences of tuberculosis (TB) are key barriers to ending TB globally. We evaluated and compared stigma, depression, and quality of life (QoL) among people with TB diagnosed through active (ACF) and passive (PCF) case-finding in Nepal.

**Methods:**

We prospectively recruited adults with TB diagnosed through ACF and PCF in four districts of Nepal between August 2018 and April 2019. Participants were interviewed at 8–12 weeks (baseline) and 22–26 weeks (follow-up) following treatment initiation. TB stigma was measured using an adapted Van Rie Stigma Scale (0 = no stigma to 30 = highest stigma). Depression was measured using a locally-validated Patient Health Questionnaire (PHQ-9). Mild and major depression were indicated by  PHQ-9 scores 5–9 and ≥ 10, respectively. QoL was measured using the EuroQoL 5-Dimension 5-level (EQ-5D-5L) from 0 to 1 (optimal QoL); and self-rated health from 0 to 100 (optimal self-rated health).

**Results:**

We recruited 221 participants (111 ACF; 110 PCF) with a mean age of 48 years (standard deviation [SD] =  ± 16), of whom 147/221 (67%) were men. The mean TB stigma score was 12 (SD = 7.3) at baseline and 12 (SD = 6.7) at follow-up. The most commonly perceived elements of TB stigma at baseline were that people with TB experienced guilt (110/221, 50%) and feared disclosure outside their household (114/221, 52%). Self-rated health and EQ-5D-5L scores increased from baseline to follow-up (69.3 to 80.3, *p* < 0.001; 0.92 to 0.9, *p* = 0.009). Nearly one-third of participants (68/221, 31%) had mild or major depression at baseline. The proportion of participants with major depression decreased from baseline to follow-up (11.5% vs. 5%, *p* = 0.012). There was a moderate, significant positive correlation between depression and stigma scores (*r* = 0.41, *p* < 0.001). There were no differences found in TB stigma, self-rated health, QoL, or prevalence of mild/major depression between ACF and PCF participants.

**Conclusions:**

We found a substantial, persistent, and clustered psychosocial impact among adults with TB diagnosed through both ACF and PCF strategies in Nepal. These findings suggest an urgent need to develop effective, evidence-based psychosocial support interventions with the potential to be integrated with existing ACF strategies and routine TB service activities.

**Supplementary Information:**

The online version contains supplementary material available at 10.1186/s44263-024-00049-2.

## Background

Tuberculosis (TB) remains a major public health problem and the leading cause of death owing to a single infectious disease. In 2022, an estimated 10.6 million people developed TB, 40% of whom were never notified of National TB Programs (NTPs) and 80% of whom lived in low- and middle-income countries (LMICs) [1]. Being ill with TB and having delayed diagnosis and care are associated not only with negative economic impacts, such as catastrophic costs [2–5], but also deleterious psychological and social (herein termed psychosocial) consequences including stigma, depression, and poor quality of life [6–9]. In short, the current biomedical approach focused on TB diagnostics and therapeutics is proving insufficient to achieve the END TB strategy goal of TB elimination. Broader strategies including active case finding (ACF) and holistic, person-centered support are required [10–12].

Stigma is recognized as one of the most important challenges to accessing TB care and becoming cured, particularly among key vulnerable populations with intersectoral barriers to health. There are several different types of TB-related stigma, including anticipated, perceived, enacted, experienced, or internalized stigma. People with TB may experience anticipated or perceived stigma where they fear being devalued when diagnosed with TB or have self-stigma resulting from internalized negative messages that are associated with TB in the community. Enacted or experienced stigma occurs when people with TB experience stigmatizing behavior from families, communities, and healthcare workers [13]. Such stigmatizing behaviors or actions can be associated with social isolation, broken marriages, discrimination, non-disclosure of TB diagnosis, and depression [7, 14–16]. TB-related stigma can also hinder care-seeking behavior [17–20], delay diagnosis, and lead to adverse TB treatment outcomes including death [21]. For these reasons, stigma was cited as a key issue requiring comprehensive political, legal, and programmatic actions in the political declaration signed by member states during the United Nations High-Level Meeting on the Fight Against Tuberculosis in 2023. These actions included increased research and investment to understand and address the issues fueling TB stigma in affected communities [22].

TB and depression have a bidirectional relationship [23, 24]. A meta-analysis reported a pooled estimated prevalence of depression among people with TB of 45.2% [25]. Another meta-analysis found that people with TB and depressive symptoms had a three-fold, four-fold, and nine-fold increased risk of death, adverse TB treatment outcomes, and loss to follow-up respectively [8]. It is clear that TB and depression are syndemic and compound negative socioeconomic and health outcomes among those affected [23, 26, 27].

Stigma, depression, and quality of life (QoL) are known to be intricately related. There is evidence that depression, stigma, and low QoL exhibit clustering at the individual level [28, 29], which can, collectively, have an adverse effect on TB treatment adherence and outcomes [30]. Stigma can be a precursor to depression and having depression can impede individuals’ capacity to engage in daily activities and mitigate stigmatizing thoughts and actions [28, 30, 31]. QoL is lower in people with TB than those without, for multifactorial reasons which include a complex interplay of physical illness, financial burden, and psychosocial consequences of the disease. Lower quality of life at treatment initiation can predict adverse TB treatment outcomes [32]. Therefore, understanding these complex consequences of TB together can elucidate the drivers and the magnitude of the impact on treatment outcomes. This new knowledge will inform the design of improved person-centered approaches to TB care delivery which are recognized as an essential component of the WHO End TB Strategy goals [22].

ACF programs involve systematic screening for TB, often among targeted high-risk or multiple disadvantaged groups such as close contacts, poor and marginalized populations, and drug users outside of healthcare settings, in order to promptly diagnose people with TB and link them to care [33]. ACF represents an opportunity for early evaluation and intervention to address the psychosocial and economic needs of people with TB to improve their health and broader outcomes, including QoL. Nevertheless, this opportunity is often missed during the implementation of existing ACF programs, which rarely include psychosocial and economic indicators and instead commonly focus on a single outcome indicator: the yield of additional TB cases.

Nepal is a lower–middle-income country in South Asia where the prevalence of TB is 416 cases per 100,000 population [34]. The prevalence is similar to other high TB burden countries including India, Bangladesh, and Pakistan [1]. Due to the high prevalence of both TB and multidimensional poverty in Nepal, the psychosocial consequences of TB are likely to be more severe in groups with lower socioeconomic status [35]. These effects are also further amplified in groups with intersectional disadvantages resulting from factors such as ethnicity, caste, multimorbidity including HIV, diabetes, and undernutrition, and historical marginalization [1, 36, 37]. Our previous research in Nepal has shown that people with TB diagnosed through ACF incurred lower TB-related direct costs during pre-treatment and intensive periods than those diagnosed through passive-case finding (PCF) [4, 5]. However, the impact or potential impact of these existing ACF programs on psychosocial indicators such as stigma, depression, and quality of life, remains unknown. The psychosocial impacts of TB could be hypothesized to be higher among people diagnosed via ACF strategies because ACF interventions usually reach people with lower socioeconomic status and barriers to healthcare access [5]. Therefore, ACF programs could be an ideal opportunity to integrate psychosocial interventions for those most vulnerable to the severe psychosocial consequences of TB. Moreover, while a handful of qualitative studies have explored stigma, depression, and quality of life as barriers to TB care in Nepal [14, 38, 39], there have been no quantitative studies examining the longitudinal psychosocial impact of TB in people with TB diagnosed through ACF and PCF.

We aimed to fill this knowledge gap to inform the development of an integrated psychosocial and economic intervention to support TB-affected households in Nepal.

## Methods

### Study design

This was a prospective longitudinal cohort study to evaluate the economic and psychosocial consequences of TB in Nepal [40]. It was nested within the larger IMPACT TB project (www.impacttbproject.org,) to generate evidence on community-based ACF models in Nepal [4, 5, 41]. From July 2017 to June 2019, the IMPACT TB study implemented community-based ACF in the study site districts including social contact tracing and mobile case-finding camps using rapid molecular testing with GeneXpert or smear microscopy [4, 5]. People with pulmonary bacteriologically confirmed, drug-sensitive TB diagnosed through ACF were defined as ACF participants. People who self-presented to public TB services in the study site districts without having received any ACF or other community-based TB outreach activities, and who were subsequently diagnosed with drug-sensitive pulmonary TB and notified to the Nepal National TB Programme (NTP), were defined as PCF participants [4]. For both ACF and PCF participants, the Nepal NTP delivered a 6-month treatment regimen for drug-sensitive TB by daily Directly Observed Treatment Short-course (DOTS) at government TB health centers [42]. The detailed process of participant selection and recruitment to IMPACT TB has been published elsewhere [5].

Between April 2018 and January 2019, a sub-sample of IMPACT TB ACF participants and unmatched PCF participants were consecutively recruited to a longitudinal cohort study and completed additional interviews during their treatment about the economic and psychosocial consequences of TB. A follow-up of this nested cohort was completed in October 2019 and the findings of the economic consequences of TB, including catastrophic costs, are reported elsewhere [5].

### Setting

The study was implemented in Nepal by a well-established and TB-focused Nepalese non-government organization, Birat Nepal Medical Trust, in the Chitwan, Mahottari, Dhanusha, and Makwanpur districts of Nepal.

**Fig. 1:**
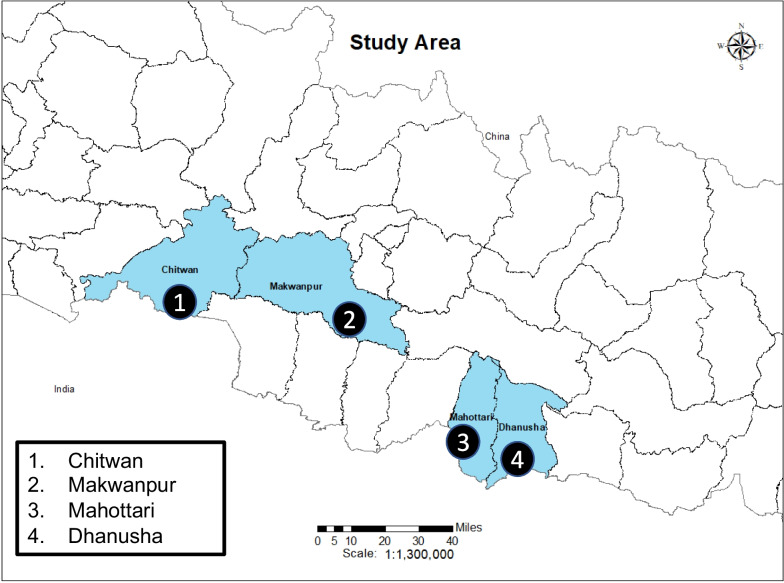
Study site districts in Nepal

These districts all share high TB burden and poverty levels but are geographically diverse: Chitwan, Mahottari, and Dhanusha districts are plains regions sharing a border with India; Makwanpur is a mid-hill district with a remote population and poor road and transportation infrastructure [43] (Fig. 1).

### Eligibility criteria

Eligible participants were adults aged 18 years or above with drug-sensitive bacteriologically confirmed drug-sensitive pulmonary TB who were recruited to the IMPACT TB project between April 2018 and January 2019 and diagnosed by either ACF or PCF [5]. People with rifampicin-resistant or multidrug-resistant TB or those diagnosed and/or treated in the private sector were not eligible to participate in the IMPACT TB cost survey due to limited time and budget availability and therefore also excluded from this study.

### Procedures and data sources

The data were collected longitudinally using an adapted, piloted, and validated version of the WHO TB Patient Cost Survey [5] with additional exploratory questions on perceptions of TB stigma, depression, and quality of life [40]. Community Health Workers (CHWs) from the IMPACT TB project administered the survey in the local language using face-to-face interview techniques during participant household visits at 8–12 weeks (herein termed “Baseline”) and 22–26 weeks (herein termed “Follow-up”) following treatment initiation.

Before obtaining a participant’s signature (or thumbprint) to indicate consent, interviewers verbally read the contents of the patient information sheet and consent form and provided an opportunity for participants to ask questions. Participants were invited to have another person of their choosing present to witness the consent, or to discuss the study with other family members before granting consent. In place of signature, participants were able to provide a thumbprint to give consent, a common and legally recognized form of signature to documents in Nepal.

### Variables and measurements

The study used measures of TB stigma, depression, and QoL as outcomes of interest to estimate the psychosocial impact of TB among participants [44]. To contextualize and evaluate the differences in levels of TB stigma, depression, and quality of life among participants between baseline and follow-up and between ACF and PCF participants, we used the minimal clinically important difference (MCID) [45, 46]. MCID is used to report the nominal detectable change in the outcome of interest that is considered to be significant or meaningful in clinical settings [47]. Where MCID was not available in the published literature, we followed Redwood et al.’s pragmatic analysis strategy of calculating the MCID as 1.96 times the standard error of the measurement [44].

#### Stigma

Stigma was measured by adapting the validated Van Rie Stigma Scale to the Nepali context [48]. The questionnaire comprised ten questions with responses on a 5-point Likert Scale of “strongly disagree” (assigned 0 points), “disagree” (assigned 1 point), “neither disagree nor agree” (assigned 1.5 points), “agree” (assigned 2 points), and “strongly agree” (assigned 3 points). The “neither agree nor disagree” response and the associated 1.5 points assigned were not included in the original Van Rie Stigma Scale but were perceived by our project team to be necessary to assess the utility of the scale in Nepal and ensure participants were not obliged to give a polarized response. Total stigma scores ranged from 0 (no stigma) to 30 (highest levels of stigma). We used the only published data on the MCID stigma scale from Redwood et al., which estimated it to be 4.30 in a sample of 84 people with DS-TB and 315 people with DR-TB in Vietnam [44]. In addition to summarizing the stigma score as a continuous variable, we also summarised stigma as a binary indicator of the proportion of participants responding “agree” or “strongly agree” vs “neither agree nor disagree”, “disagree”, and “strongly disagree” for each stigma scale domain.

In this study, the project team initially reviewed the Van Rie Stigma Scale together. We opted to combine questions about the “community perspectives towards TB” and “patient perspectives towards TB” for multiple reasons. First, the project team wanted to measure perceptions of stigma at both the individual level and community level. Second, this study was part of a larger longitudinal study evaluating the health, psychosocial, and economic impact on people with TB diagnosed through ACF and PCF. In the planning stages, the team was cognizant of the length of the survey and wanted to balance robust data collection with a reduction in participant time and responder fatigue; in addition, first-person questions (“I feel…”, “I experience….”) were felt by the project team to be overly sensitive, probing, and with the potential to cause distress in the study site settings. To address these issues, we purposefully selected third-person (“Some people in the community…”, “Some people with TB….”) stigma questions, which we felt to be most socio-culturally pertinent and sensitive at the individual and community level in Nepal, to also facilitate a more concise section of the survey. The questions were initially employed among 10 participants, and tested for clarity and completeness.

#### Depression

Depression was measured using an adapted version of the Patient Health Questionnaire (PHQ-9), which has been validated in Nepal [49, 50]. The adapted tool uses an initial screening question about low mood, which translates literally in English to “Have you experienced heart-mind problems over the past 2 weeks?”. In Nepali, the term is known as ‘*manko samasya’* which relates to the problem in the organs of emotion (heart) and memories (mind) and includes conditions such as sadness and negative memories [50]. Participants answering “No” are not asked any further questions and are presumed to have a PHQ-9 score of 0. Participants answering “Yes” go on to answer the full PHQ-9 questions related to symptoms of depression. We included eight PHQ-9 questions. During the piloting of the questionnaire, the study team reached a consensus that the question “How often in the past 2 weeks have you been having bad thoughts about your own self, feeling like being responsible for your own failure or having let your family down?” could cause distress for participants in the local context. Therefore, the question was not administered during implementation due to the lack of mental health services to provide further expert counseling with qualified mental health professionals being rare outside of Kathmandu. Possible responses were on a four-point scale of “0: not at all”, “1: sometimes”, “2: usually”, and “3: always”. Total PHQ-9 score in this study ranged from 0 (no depression) to 24 (highest depression). As has been demonstrated to be highly sensitive and specific in other settings, the scores were categorized into no depression (scores between 0 and 4), mild depression (scores between 5 and 9), and major depression (scores ≥ 10 and above) [49]. Based on the relevant available literature, the MCID for the PHQ-9 scale was five [46].

#### Quality of life

To assess the quality of life, we adapted the EQ-5D-5L index tool, which uses five dimensions of quality of life: mobility, self-care, usual activities, pain or discomfort, and anxiety or depression and a visual analog scale that includes a self-health rating [51]. Each question includes a five-category response scale from 1: no problem, 2: slight problem, 3: moderate problem, 4: severe problems, 5: being unable. Since an existing set of scores were not available for Nepal, we applied the sets from the most geographically and socioculturally proximate country, India, and totaled to get a utility score, from 0 (lowest quality of life) to 1 (highest quality of life) [52]. Nepal and India are among the 30 high TB burden countries with a high prevalence of stigma, poverty rates, and low literacy. Other published value sets either represented high or middle-income countries and therefore were not appropriate for our study. Similarly, the health rating was measured on a visual analog scale of 0 to 100 based on participants’ self-reported health. The participants were asked to label an appropriate point that best defined their health condition that day with a mark on the scale printed in the questionnaire with 0 indicating the worst health and 100 indicating the best health of the participant [51]. Despite a thorough review of the literature, no evidence of MCID estimates relating to EQ-5D-5L among people with TB was identified. Therefore, we used an MCID from a study on people with TB in South Africa which used the EQ-5D-3L index for people with TB as 0.07 [53]. The questions were initially employed among 10 participants, and tested for clarity and completeness.

### Sample size calculation and sampling

There was no formal sample size calculation for this study on the psychosocial impact of TB. The participant sample size was calculated for the TB Patient Cost Survey performed within the IMPACT-TB project, which was based on the prevalence of catastrophic costs among TB-affected households identified by ACF vs PCF. Previous TB PCS have suggested that a sample size of 100 people with TB and their households provides a representative distribution of TB-affected household-level costs [54–56]. To compensate for potential attrition, we aimed to consecutively recruit 110 PCF and 110 ACF participants respectively during the study period. The sample size of 220 participants, 110 PCF and 110 ACF was sufficient to detect a mean difference of 3.7 in levels of stigma, 1.5 in levels of depression, and 0.10 in quality of life with a power of 0.8 and an alpha value of 0.05.

### Statistical methods

The study adhered to the STrengthening the Reporting of OBservational studies in Epidemiology (STROBE) guidelines [57]. The study used descriptive statistics to summarize the participants’ socio-demographic details, comorbid conditions, depression, stigma, and quality of life and compare these data at baseline, follow-up, and the change over time for ACF participants, PCF participants, and the overall cohort. The proportion of missing responses to each question on psychosocial impact including TB stigma, depression, and quality of life, was calculated. Van Rie stigma scale domain responses shown in the supplementary files, demonstrated full completion of stigma questions with no missing responses and < 4% of responses being “neither agree nor disagree”. Similarly, the full responses to each PHQ-9 question, shown in the Additional file 1: Table S2, showed high rates of completion with 2/221 (0.9%) and 4/221 (1.8%) of participants not fully completing the PHQ-9 at baseline and follow-up respectively.

Continuous data were summarised by mean and standard deviation and compared: ACF vs PCF participants at baseline, follow-up, and change from baseline to follow-up using the two group Student’s *t*-test; and change from baseline to follow-up among ACF participants, among PCF participants, and all participants using the Repeated Measures ANOVA test. Discrete variables that depict sociodemographic and clinical characteristics were expressed in proportion and percentage and compared between ACF and PCF participants at baseline, follow-up, and the change between baseline and follow-up using the chi-square test. Spearman’s coefficient was used to evaluate the correlation between stigma and depression scores and the *p* value of the correlation had Bonferroni adjustment. Data were analyzed using Stata Version 15. *P* values of < 0.05 were considered statistically significant.

## Results

### Demographic characteristics

Of 221 people invited, 100% were recruited and completed the survey, of whom 111 and 110 were diagnosed through ACF and PCF strategies respectively (Table 1). The mean age was 48 years (SD =  ± 16.0) and two-thirds (67%) of participants were male, consistent with the known gender ratio of TB cases in Nepal. Over half of the participants (54%) were illiterate and without formal education, of whom 46% were female. Almost 40% were unemployed at baseline. Comorbid conditions were reported among 11% of participants. ACF participants had lower education levels, were poorer, and were more likely to live in crowded housing than PCF participants (Table 1).

**Table 1:** Sociodemographic and clinical characteristics of participants at baseline

Characteristics	ACF	PCF	Total	*p* value^*^
	(*n* = 111) *n* (%)	(*n* = 110) *n* (%)	(*N* = 221) *n* (%)	
Sex				
Male	71 (64)	76 (69)	147 (67)	0.42
Female	40 (36)	34 (31)	74 (33)	
Age (years)				0.15
18–35	21 (19)	33 (30)	54 (24)	
36–55	43 (39)	39 (35)	82 (37)	
≥ 56	47 (42)	38 (35)	85 (39)	
Age, mean (SD)	50 (15)	46 (17)	48 (16)	0.057
Education level completed				**0.029**
No education and illiterate	69 (62)	50 (45)	119 (54)	
Basic literacy (able to read and write)	14 (13)	13 (12)	27 (12)	
Basic school	18 (16)	24 (22)	42 (19)	
Secondary and higher education	10 (9)	23 (21)	33 (15)	
Occupation				0.073
Farmer	23 (21)	16 (15)	39 (18)	
Labourer	14 (13)	5 (4)	19 (9)	
Salaried employee	8 (7)	15 (13)	23 (10)	
Unemployed	40 (36)	49 (45)	89 (40)	
Others	26 (23)	25 (23)	51 (23)	
Crowding				
Crowded	55 (50)	31 (28)	86 (39)	**0.001**
Not crowded	56 (50)	79 (72)	135 (61)	
Smoking				
Ever smoked	58 (52)	53 (48)	111 (50)	0.55
Never smoked	53 (48)	57 (52)	110 (50)	
Alcohol				
Ever drunk alcohol	57 (51)	46 (42)	103 (47)	0.16
Never drunk alcohol	54 (49)	64 (58)	118 (53)	
Poverty level				
Lowest tercile (poorest)	57 (51)	31 (28)	88 (40)	**0.002**
Middle tercile	33 (30)	46 (42)	79 (36)	
Highest tercile (least poor)	21 (19)	33 (30)	54 (24)	
Comorbidities				
No comorbidities	97 (87)	87 (79)	184 (83)	0.099
At least one comorbidity^*^	14 (13)	23 (21)	37 (17)	

^*^Comorbidities included diabetes (*n* = 14), obstructive pulmonary disease (*n* = 7), hypertension (*n* = 5), chronic arthritis (*n* = 3), HIV (*n* = 2), gastrointestinal disease (*n* = 2), cancer (*n* = 1), blindness (*n* = 1), chronic kidney disease (*n* = 1), and thyroid disease (*n* = 1)

### The psychosocial impact of TB

Table 2 summarizes TB stigma scores, PHQ-9 scores, EQ-5D-5L scores, and self-rated health at baseline and follow-up and compares the change from baseline to follow-up among all participants, ACF participants, and PCF participants. Table 3 summarises and compares the baseline, follow-up, and change between baseline and follow-up TB stigma scores, PHQ-9 scores, and EQ-5D-5L scores, among ACF vs PCF participants.

**Table 2:** Comparison of stigma, depression, and quality of life changes between baseline and follow-up

Scales	Max Score	MCID of score	All (*N* = 221)				ACF (*n* = 111)				PCF (*n* = 110)			
			Baseline	Follow-up	Change	*p* value	Baseline	Follow-up	Change	*p* value	Baseline	Follow-up	Change	*p* value
Stigma score (SD)	30	4.3	12.0 (7.3)	12.0 (6.7)	0.0 (5.7)	0.96	11.7 (7.9)	11.6 (7.4)	-0.15 (6.4)	0.8	12.2 (6.6)	12.3 (5.9)	0.11 (4.9)	0.81
PHQ-9 (SD)	24	5	2.9 (4.4)	2.4 (4.0)	− 0.5 (4.5)	0.07	3.3 (4.7)	2.0 (3.8)	− 1.2 (4.4)	**0.004**	2.6 (4.2)	2.8 (4.1)	0.15 (4.5)	0.73
QoL (SD)	1	0.07	0.92 (0.18)	0.97 (0.07)	0.05 (0.16)	**0.009**	0.91 (0.22)	0.96 (0.09)	0.05 (0.19)	**0.003**	0.94 (0.13)	0.98 (0.04)	0.04 (0.11)	**0.002**
Self-rated health	100	N/A	69.3 (15.0)	80.3 (13.6)	11 (17.1)	** < 0.001**	67.8 (16.0)	78.9 (14.5)	11.1 (18.3)	** < 0.001**	70.9 (13.8)	81.7 (12.5)	10.8 (16)	** < 0.001**

*ACF* active case finding, *PCF* passive case finding, *PHQ-9* Patient Health Questionnaire, *QoL* quality of life. *P* values represent difference in stigma score, PHQ-9, QoL, and self-rated health from baseline to follow-up amongst all participants, ACF participants, and PCF participants tested using the repeated measures ANOVA test

**Table 3:** Stigma, depression, and quality of life at baseline and follow-up amongst ACF vs PCF participants

Scales	Max score	MCID of score	Baseline			Follow-up			Change from baseline to follow-up		
			ACF *n* = 111	PCF *n* = 110	*p* value	ACF *n* = 111	PCF *n* = 110	*p* value	ACF *n* = 111	PCF *n* = 110	*p* value
Stigma score (SD)	30	4.3	11.7 (7.9)	12.2 (6.6)	0.65	11.6 (7.4)	12.3 (5.9)	0.43	− 0.15 (6.4)	0.11 (4.9)	0.73
PHQ-9 (SD)	24	5	3.3 (4.7)	2.6 (4.2)	0.29	2.0 (3.8)	2.8 (4.1)	0.16	− 1.2 (4.4)	0.15 (4.5)	**0.02**
QoL (SD)	1	0.07	0.91 (0.22)	0.94 (0.13)	0.2	0.96 (0.09)	0.98 (0.04)	0.09	0.05 (0.19)	0.04 (0.11)	0.47
Self-rated health	100	N/A	67.8 (16.0)	70.9 (13.8)	0.12	78.9 (14.5)	81.7 (12.5)	0.12	11.1 (18.3)	10.8 (16)	0.9

*ACF* active case finding, *PCF* passive case finding, *PHQ-9* Patient Health Questionnaire, *QoL* Quality of Life. *P* values represent the difference in stigma score, PHQ-9, QoL, and self-rated health in ACF vs PCF participants at baseline, follow-up, and change from baseline to follow-up tested using two-group Student’s *t*-test

### Stigma

The mean TB stigma scores at baseline and follow-up for all participants were 12.0 (SD = 7.3) and 12.0 (SD = 6.7), respectively, with a change from baseline to follow-up of 0.0 (SD = 5.7, Table 2). There were no significant differences or differences greater than the MCID threshold found in the change between baseline and follow-up TB stigma scores of all participants, ACF participants, and PCF participants (Table 2) or in the change between baseline and follow-up TB stigma scores of ACF vs PCF participants (Table 3).

**Fig. 2:**
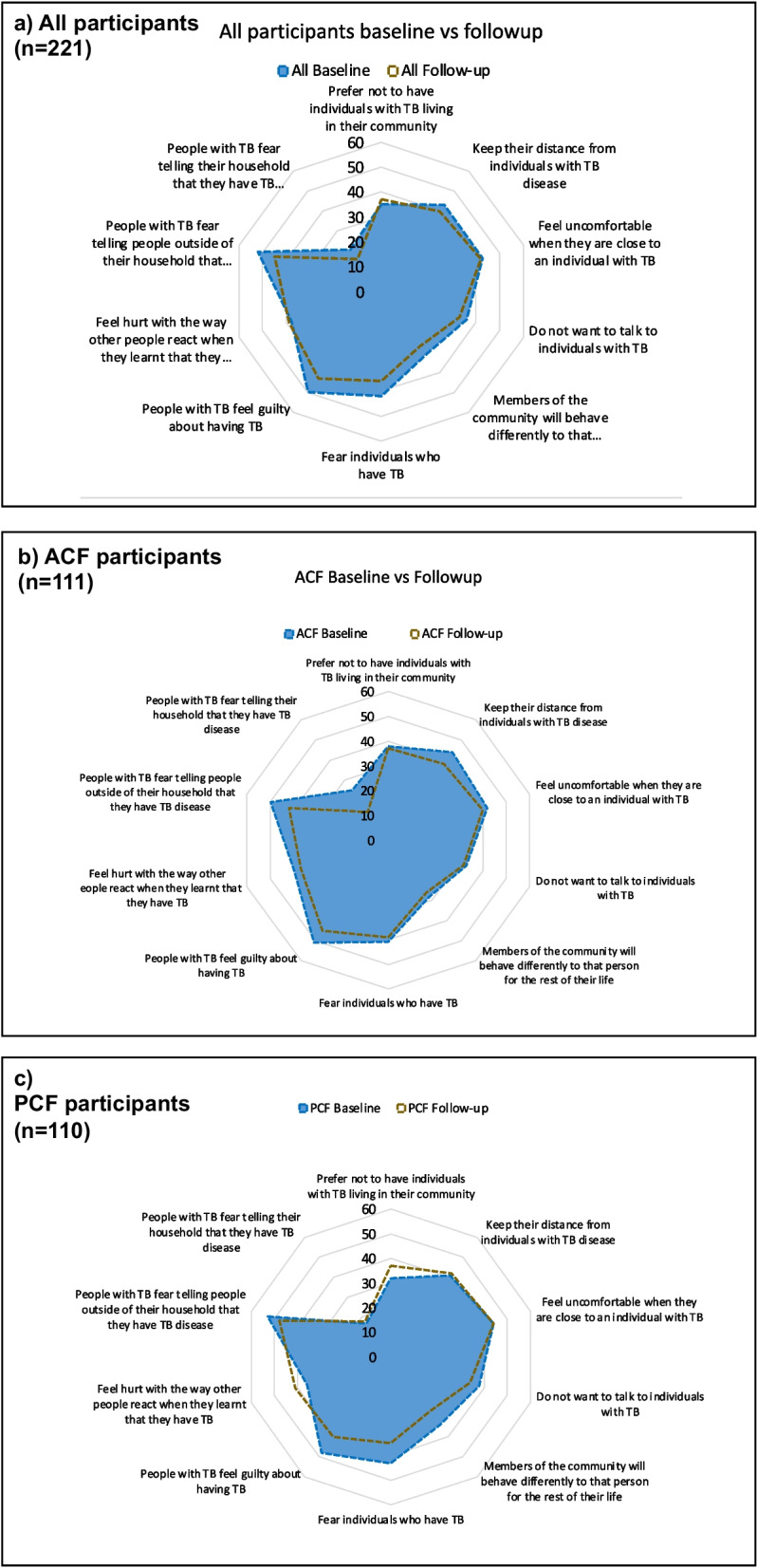
The blue color represents the percentage of people reporting ‘strongly agree’ or ‘agree’ with the items of the Van Rie stigma scale at the baseline period. The orange-brown color represents the percentage of people at the baseline period reporting ‘strongly agree’ or ‘agree’ with the items of the Van Rie stigma scale for the follow-up period of the study

With regards to specific items from the TB stigma scale at baseline, a sizeable proportion of participants reported that people with TB experienced guilt and fear of disclosure: 110/221 (50%) participants agreed ‘some people with TB feel guilty about having TB’; and 114/221 (52%) agreed ‘some people with TB fear telling people outside of their household’. A fifth of participants 47/221 (21%) agreed that ‘some people with TB fear telling their household that they have TB disease’ (Fig. 2a), with more men (72%) than women (28%) in agreement. Between baseline and follow-up, among all participants (Fig. 2a), ACF participants (Fig. 2b), and PCF participants (Fig. 2c), there was minimal reduction in the prevalence of TB stigma across most of the 10 items of the scale. Among PCF participants, the proportion of participants who agreed with questions including ‘Some people prefer not to have individuals with TB living in their community’, ‘Some people keep their distance from individuals with TB disease’, ‘Some people feel uncomfortable when they are close to an individual with TB’, and ‘Some people with TB feel hurt with the way other people react when they learn they have TB, did not change between baseline and follow-up (Fig. 2c). Detailed participants’ responses reporting the Van Rie stigma scale at baseline and follow-up for ACF and PCF participants are presented in Additional file 1: Table S1.

#### Depression

Among all participants, 70/221 (32%) and 64/221 (29%) had a positive PHQ-9 depression screening question at baseline and follow-up, respectively, and went on to complete the remaining PHQ-9 questions (Additional file 1: Table S2). The mean PHQ-9 depression scores of all participants were 2.9 (SD = 4.4) and 2.4 (SD = 4.0) at baseline and follow-up, respectively (*p* = 0.07, Table 2).

Depression scores decreased significantly between baseline and follow-up among ACF participants (3.3 [SD = 4.7] baseline vs 2.0 [SD = 3.8] follow-up, *p* = 0.004) but not among PCF participants (2.6 [SD = 4.2] vs 2.8 [SD = 4.1], *p* = 0.73, Table 2). Changes in depression scores between baseline and follow-up were more pronounced among ACF participants than PCF participants, (change − 1.2 [SD = 4.4] vs 0.15 [SD = 4.5], *p* = 0.02, Table 2).

There were no differences greater than the MCID threshold found in the change between baseline and follow-up depression scores of all participants, ACF participants, and PCF participants (Table 2) or in the change between baseline and follow-up depression scores of ACF vs PCF participants (Table 3).

When the scores were categorized into three levels (no depression, mild depression, and major depression), we found nearly one-third of participants (68/221, 31%) had some form of depression at baseline, with 43/221 (19.5%) having mild and 25/221 (11.5%) major depression (Table 4). Among all participants, there were significant changes in the proportional distribution of no, mild, and major depression between baseline and follow-up (*p* = 0.012, Table 4). The proportion with major depression decreased (25/221 [11.5%] at baseline vs 11/221 [5%] at follow-up) and there was a concomitant increase in those with mild depression (43/221 [19.5%] vs 49/221 [22%]) and no depression (153/221 [69%] vs 161/221 [73%], Table 4). Changes in the proportional distribution of no, mild, or major depression did not reach significance when analyzed by sub-group of ACF (*p* = 0.067) or PCF (*p* = 0.084) diagnosis (Table 4).

**Table 4:** Comparison of the changes between baseline and follow-up of no/mild/major depression among participant groups

Depression category by PHQ-9 score	All (*N* = 221)				ACF (*n* = 111)				PCF (*n* = 110)			
	Baseline	Follow-up	Change	*p* value	Baseline	Follow-up	Change	*p* value	Baseline	Follow-up	Change	*p* value
No depression, *n* (%) [0–4]	153 (69)	161 (73)	8 (4)	**0.012**	74 (67)	86 (77)	12 (10)	0.067	78 (72)	75 (68)	-4 (-4)	0.084
Mild depression, *n* (%) [5–9]	43 (19.5)	49 (22)	6 (3)		21 (19)	18 (16.5)	− 3 (− 3)		23 (20)	31 (28)	9 (8)	
Major depression, *n* (%) [≥ 10]	25 (11.5)	11 (5)	− 14 (-6)		16 (14)	7 (6.5)	− 9 (-8)		9 (8)	4 (4)	− 5 (− 5)	

*P* values are derived from chi-squared tests comparing the change between baseline and follow-up in the percentages of all, ACF, and PCF participants with no depression, mild depression, and major depression

There were no significant differences found between the proportion distribution of no, mild, or major depression among ACF vs PCF participants at baseline, follow-up, or change between baseline and follow-up (Table 5).

**Table 5:** Comparison of percentage of ACF vs PCF participants with no/mild/major depression at baseline and follow-up

Depression category by PHQ-9 score	Baseline			Follow-up			Change from baseline to follow-up		
	ACF (*n* = 111)	PCF (*n* = 110)	*p* value	ACF (*n* = 111)	PCF (*n* = 110)	*p* value	ACF (*n* = 111)	PCF (*n* = 110)	*p* value
No depression [0–4] (%)	74 (67)	78 (72)	0.34	86 (77)	75 (68)	0.08	12 (10)	− 4 (− 4)	0.73
Mild depression [5–9] (%)	21 (19)	23 (20)		18 (16.5)	31 (28)		− 3 (− 3)	9 (8)	
Major depression [≥ 10] (%)	16 (14)	9 (8)		7 (6.5)	4 (4)		− 9 (− 8)	− 5 (− 5)	

*P* values are derived from chi-squared tests comparing the percentage of ACF participants vs PCF participants with no depression, mild depression, and major depression at baseline, follow-up, and the change between baseline and follow-up

Among those who screened positive for depression, there was a moderate, significant positive correlation between depression scores and stigma scores at baseline (*r* 0.41, *p* < 0.001) but no association at the follow-up (*p* = 0.9). (Additional file 2: Figure S1) The most prevalent issues reported in the disaggregated PHQ-9 responses included lack of appetite and energy (Additional file 1: Table S2).

#### Quality of life

At baseline, 3% of the participants reported having severe problems or being unable to walk or self-care; approximately 2% of participants reported having severe problems or being unable to do usual activities and 4% reported having anxiety. Similarly, at baseline, among ACF and PCF participants, a higher proportion of participants from the ACF group reported having severe problems or being unable for four dimensions except pain or discomfort. At follow-up, less than 1% reported having severe problems or being unable for all the five dimensions of EQ-5D-5L. Similarly, none of the PCF participants reported having severe problems or being unable to for the four dimensions except anxiety and depression (Additional file 1: Table S3).

The self-reported mean health rating for all participants improved by 11.3 points from 69.0 (SD = 15.0) at baseline to 80.3 (SD = 13.6) at follow-up (*p* < 0.001, Table 2). The mean EQ-5D-5L score for all participants increased by 0.05 from 0.92 (SD = 0.18) at baseline to 0.97 (SD = 0.07, *p* = 0.009) at follow-up. Among both ACF and PCF groups, self-reported health rating (both *p* < 0.001) and EQ-5D-5L scores (ACF *p* < 0.003; PCF *p* = 0.002) increased between baseline and follow-up (Table 2). However, there were no significant differences in self-reported health rating or EQ-5D-5L among ACF vs PCF at baseline, follow-up, or the change between baseline and follow-up (Table 3). There were no differences greater than the MCID threshold found in the change between baseline and follow-up EQ-5D-5L and self-rated health scores of all participants, ACF participants, and PCF participants (Table 2) or in the change between baseline and follow-up EQ-5D-5L and self-rated health scores of ACF vs PCF participants (Table 3).

The disaggregated data for the responses across the five domains of the EQ-5D-5L are shown in the Additional file 1: Table S3.

## Discussion

To our knowledge, this is the first study to measure the perception of TB stigma, depression, and quality of life longitudinally among people with TB and to compare these between people diagnosed with TB through ACF and PCF. Nearly one-third of participants had mild or major depression at baseline. The proportion of participants with major depression decreased between baseline and follow-up and, amongst ACF but not PCF participants, the depression score decreased between baseline and follow-up. Most of the items on the stigma scale decreased at follow-up for both ACF and PCF participants. TB stigma and depression scores were positively correlated. Self-reported quality of life and EQ-5D-5L scores improved between baseline and follow-up for all participants but no differences in quality of life were found between ACF and PCF participants. These findings emphasise the stark psychosocial consequences of TB and highlight ACF activities as an early point-of-contact with vulnerable people with TB, which currently represents a missed opportunity to intervene and improve psychosocial outcomes.

### Stigma

There is strong evidence that TB remains a severely stigmatized disease, especially within high-burden communities in LMICs [19]. In this cohort of people with TB in Nepal, TB stigma, especially related to guilt and fear of disclosure outside of the household, was prevalent and persistent throughout TB treatment, regardless of whether participants were diagnosed through ACF or PCF. This finding contradicts other studies that reported a reduction in TB stigma over time, more often at the continuation phases of TB treatment. For example, cross-sectional studies conducted in Ethiopia among people with TB found that individuals in the intensive phase of TB treatment were more likely to report TB stigma than those in the continuation phase [58, 59]. Longitudinal studies on stigma associated with diseases such as HIV also have shown that stigma, particularly internalized stigma is higher following diagnosis and gradually decreases over time [60]. Zambian and South African TB and HIV Reduction (ZAMSTAR) study also showed a reduction in levels of internalized stigma over the treatment course [61]. This is also in concordance with an article by Earnshaw et al. that describes how people may be able to overcome their stigmatized status over time when the severity of symptoms begins to gradually wane [62]. Interventions that aim to reduce stigma during the early stages of treatment are therefore important for curable diseases like TB. However, there is a paucity of evidence globally and in Nepal that determines the underlying cause for the persistence of TB-related stigma in communities, and warrants future research.

In our longitudinal cohort, we did not find significant differences in the stigma scores between our baseline interview conducted during the intensive phase and our follow-up interview conducted during the continuation phase. Nevertheless, we found a high proportion of participants reporting TB stigma, including guilt and fear of disclosure, which persisted throughout both phases. This is important because TB-related stigma has been shown to contribute to diagnostic or treatment delay, non-adherence to TB medications, and adverse TB treatment outcomes [19, 21]. There were no differences between TB stigma scores among participants diagnosed through ACF or PCF at any time point or across time points. Although ACF represents a prime opportunity to promptly identify and address stigma among TB-affected households, it is notable that TB stigma has also been found to be a barrier to the successful implementation of ACF programs [63–65]. This should be taken into account in the design and implementation of any ACF program aiming to incorporate TB stigma reduction activities within the interventions.

The 10-item adapted TB stigma scale had very low non-response or equivocal response rates. Half of the cohort was in agreement that people with TB fear telling people outside their household about their disease status. Of perhaps greater concern, a fifth of participants agreed that people with TB fear even telling people within their own household about their illness, which reflects the highly stigmatized nature of TB in Nepali society. It is well recognized that people with TB report hiding their illness [7, 66, 67] and that such non-disclosure is associated with stigma, fear and isolation, worsening depression, delayed diagnosis, developing drug-resistant TB, and sustained transmission [31, 68–71]. Our findings are concurrent with other studies that found that people with TB are more likely to disclose their disease to people within their household than non-household members, although more men than women in our cohort reported fear of disclosure within their households [68, 72]. The perception that people with TB feel guilt due to their disease which was prevalent among our cohort, has been reported in other studies including a cross-sectional study in Kathmandu Valley, Nepal [7, 31, 73].

Based on our findings related to TB stigma, we have since developed and piloted a complex psychosocial and economic intervention, a component of which aims to support people to recognize, cope, and challenge the stigma associated with TB [74]. The stigma intervention is a locally made animated video about TB stigma, which is shown during household visits and at mutual support “TB Clubs”, which are knowledge- and experience-sharing events led by TB survivors to which all TB-affected household members are invited [75]. We will evaluate the effect of this video on the major stigma domains identified in our study, enhance pre-existing emotional support of the affected person by their household and inform the design of TB stigma reduction intervention in communities.

### Depression

The intensive phase of TB treatment, nearly one-third of our cohort had mild (20%) or major (12%) depression, which is in line with the existing body of evidence of an association between TB and depression [23, 25]. Similarly, comparable to a study in Malaysia, our study showed depression persisted even at the end of TB treatment [76]. A study by Ambaw et al. showed untreated depression at the initiation of treatment, is associated with poor quality of life and increased disability at the end of treatment [77]. We also found that, among people with mild or major depression, depression scores correlated positively with stigma scores, suggesting a clustering of the psychosocial consequences of TB. Our study adds to the scant literature on the longitudinal measurement of depression: in our cohort, the distribution of no, mild, and major depression changed significantly between the baseline and follow-up interviews, with major depression decreasing by more than half. This suggests that earlier psychosocial support interventions combined with stigma reduction programs could be effective in reducing the impact of TB on mental health and internalized stigma. A similar reduction in the magnitude of depression was seen in two longitudinal studies conducted in India [78, 79]. Rouf et al. also reported that people with TB who experienced depression persisting after the intensive phase had a higher likelihood of adverse TB treatment outcomes including treatment failure [79]. An analysis of 48 LMICs showed that having comorbid depression and TB was associated with an increase in problems related to sleep, self-care, mobility, and pain [23], all measures of quality of life.

In our study, the prevalence of depression among people with TB at baseline (end of intensive phase) and follow-up (end of continuation phase) was higher than other cross-sectional studies conducted in Nepal (10%) and China (18%) but similar to Nigeria (28%) and Vietnam (25%) [44, 80–82]. The differences in depression prevalence across countries may relate to the time point during TB treatment at which the depression scale was applied and also to sociodemographic differences including urban or rural location, age, gender, inadequate social support, and low education status [83]. For example, the majority of participants in our study were aged 55 years and above, an age group which has been shown in Ethiopia and Nigeria to have higher rates of depression compared to younger age groups [84, 85]. Nevertheless, our findings of prevalent depression among people with TB coupled with a lack of integrated mental health screening programs in Nepal represent a significant challenge for Nepal’s NTP [86].

We found a significant decrease in depression scores between baseline and follow-up among ACF participants. This could be due to increased contact, support, and informal counseling from experienced community health workers for those diagnosed with ACF. To our knowledge, this is the only quantitative data to evaluate the impact of ACF on depression. A previous qualitative study and scoping review reported ACF interventions benefitted people with TB through trust, good communication, and addressing fear and stigma [64, 87]. This highlights the need to strengthen resources to integrate community-proven ACF activities, such as household TB screening, with concomitant screening for and management of mental illness (such as counseling and psychological therapies) as part of routine NTP activities, to reduce depression and address mental health issues associated with TB [88, 89].

### Quality of life

We observed that the quality of life of people with TB, whether measured by EQ-5D-5L utility tool or self-rated health, improved between the intensive and continuation phases of treatment for ACF, PCF, and all participants. This is consistent with other evidence including a longitudinal study from Pakistan [9, 90]. Although our findings do not show differences in quality of life between the ACF and PCF participants, ACF has been found as an effective strategy to improve quality of life [91].

Similar to our findings, longitudinal studies conducted in Peru by Datta et al. and in Canada by Bauer et al. showed the quality of life at treatment initiation is lower than for those without TB [32, 92]. Datta et al. also found the quality of life increased to a level similar to people without TB after 6 months of TB treatment [32]. However, a study in South Africa reported that, even at the end of TB treatment, quality of life was worse for people with TB than those without TB [53]. We also observed that the EQ-5D-5L score during the intensive phase was higher among our cohort than in a study conducted in South Africa [53]. Studies have shown that family and social support for people with TB improves their quality of life [93, 94].

The proportion of participants reporting problems for the five levels in EQ-5D-5L was lower in our longitudinal study compared to that in Pakistan [90]. This could be due to the difference in the time period of the interview (2 months in Pakistan versus 2–3 months for our study) and/or the use of the EQ-5D-5L versus EQ-5D-3L tool. The time period during which quality of life is measured is important as the quality of life improves as treatment progresses and the differences in the levels of the tool may differ in the self-reported severity of the illness.

### Stigma, depression, and quality of life

Our study found that a high proportion of people with TB perceived TB stigma. The association between stigma and depression is well established and individuals having internalized or TB stigma have a higher likelihood of having depression [30]. This suggests that psychosocial support programs including stigma reduction interventions, especially when implemented early in treatment, could be effective in mitigating the impact of TB on mental health and internalized stigma. The study found that the proportion of participants with major depression decreased during the follow-up period. The reasons for this improvement during follow-up are likely to be multi-factorial, including improved physical health due to treatment, decreased financial stress on the household due to a return to income-generating activities, and reduced out-of-pocket medical expenditure compared to the diagnostic phase of the illness, decreased fear of mortality from TB, and other inter-related factors. Therefore, it is important to design comprehensive health programs that support overcoming the negative health effects of stigma and depression for people with TB.

It is also interesting to note the findings that despite the decrease in depression towards the end of treatment, stigma persisted for both ACF and PCF groups. Stigma has been associated with years of education, poor knowledge regarding TB and its transmission, perceived risks of transmission, poverty, and socioeconomic class [19, 30]. In our study, more than half of our participants did not have basic literacy and are poor which might be the reason for enduring stigma in this cohort. Thus, further study is needed to measure other factors that cause stigma and depression for people with TB and develop better people-centric TB care.

### Strengths and limitations

This is the first longitudinal study measuring TB stigma, depression, and quality of life among people with TB during TB treatment in Nepal. It is also one of the few studies that compared participants diagnosed with TB from ACF and PCF strategies. The findings will contribute to informing researchers and policymakers aiming to design and develop locally appropriate psychosocial interventions to improve the health, mental health, and quality of life of people with TB in low- and middle-income countries like Nepal.

The study has several limitations. This was a formative and opportunistic study taking part within a larger program of research including an ACF study with an associated TB Patient Costs survey. Due to logistical, time, and budgetary constraints, the study was unpowered and unmatched, which may have introduced selection bias. Indeed, by their very nature, ACF interventions are aimed at identifying people with TB who may be more vulnerable and less able to access healthcare, which was reflected in the baseline socioeconomic differences in our ACF and PCF cohort. Therefore, the finding of no difference in the psychosocial consequences of TB among ACF vs PCF participants, apart from the change in depression scores between baseline and follow-up in ACF vs PCF participants, should be interpreted with caution. To overcome this issue of selection bias, we have since completed recruitment and follow-up of the randomized-controlled “ASCOT” pilot trial of a socioeconomic intervention, including TB-stigma reduction activities, during which we longitudinally measured levels of stigma, depression, and quality of life [74]. Another limitation of our study was the use of adapted versions of PHQ-9, Van Rie Stigma Scale, and EQ-5D-5L tools. The removal, due to the issue related to the potential to cause distress identified during piloting, of a question from the PHQ-9 scale, meant the maximum possible score was 24 rather than 27. This change could have potentially resulted in a small underestimation of the prevalence and severity of depression in our cohort, underlining the main finding that depression is highly prevalent among TB cases in Nepal. At the time the study was being conducted, there were no formal mental health services available in the districts or a mechanism for referral of people identified as having major depression to mental health services further afield. Our staff used an informal referral pathway which we recognized needed to be systematised. We have since developed a referral pathway to address this issue, which has been successfully used during the subsequent ASCOT pilot trial [74]. The Van Rie Stigma Scale was also adapted to be more concise and to remove questions perceived as potentially insensitive but, in future research, we aim to formally validate the psychometric properties of the scale in Nepal, as we have done in other settings including Indonesia [95–97]. Similarly, the EQ-5D-5L tool is a simple tool appropriate for field studies, but as a consequence, it is also a rather ‘blunt’ tool for measuring physical changes in quality of life for drug-sensitive TB. We adapted our weighted EQ-5D-5L score from an Indian quality of life dataset that had used EQ-5D-3L, which may have meant contextual and measurement differences were introduced, which we did not evaluate within this study. The use of MCID values to contextualize differences in psychosocial impact was limited by a scarcity of evidence on MCIDs in this field. Nevertheless, the use of MCID values by researchers and implementers appears to be growing and they will be refined further as more robust evidence becomes available to inform the parameters [44]. Finally, there is the possibility that longitudinal administration of tools measuring the psychosocial impact of TB could have introduced desirability bias amongst participants in their responses at follow-up interviews. However, conversely, we believe the longitudinal study design would reduce recall bias compared to the standard cross-sectional studies that have been reported in this field. The longitudinal design has added value in measuring multiple psychosocial impacts of TB during the course of treatment for the first time in Nepal [31, 44, 72, 73, 98].

## Conclusions

Our findings show a substantial, persistent, and clustered psychosocial impact of TB in Nepal. One-third of people with TB had some form of depression and levels of depression were reduced amongst ACF but not PCF participants. TB diagnosis provides an opportunity to evaluate and identify people who would benefit from support through holistic psychosocial interventions, which could be integrated with ACF strategies and routine TB services.

## Supplementary Information

**Additional file 1: Table S1.** Participants responses in percentage reporting the van Rie stigma scale at baseline and follow-up. **Table S2.** Participants responses in percentage reporting for each Patient Health Questionnaire question at baseline and follow-up. **Table S3.** Participants responses in percentage reporting the experience of quality of life at baseline and follow-up.

**Additional file 2: Figure S1.** Correlation between baseline depression and stigma scores amongst participants who screened positive for depression (*n*=71).

## Data Availability

The datasets used and/or analyzed during the current study are available from the corresponding author upon reasonable request. Email: Tom.Wingfield@lstmed.ac.uk. This is because the ethical approval for the study that was received in both the UK and Nepal specified the publication of findings in peer-reviewed journals and presentation through public engagement activities and made no specific mention of making the data publicly available. This was also not stated in any study documents including the protocol, consent forms, or participant information leaflets.

## Abbreviations

ACF: Active case finding
BNMT: Birat Nepal Medical Trust
DOTS: Directly Observed Treatment Short-course
LMIC: Low- and middle-income countries
LSTM: Liverpool School of Tropical Medicine
MCID: Minimal Clinically Important Difference
NTP: National Tuberculosis Program
PCF: Passive case finding
PCS: Patient Cost Survey
PTB: People with tuberculosis
QoL: Quality of life
TB: Tuberculosis
VRSS: Van Rie Stigma Scale
