# Supplementary material for: Stigma, depression, and quality of life among people with pulmonary tuberculosis diagnosed through active and passive case finding in Nepal: a prospective cohort study
Source: BMC Glob Public Health. 2024 Mar 24;2:20. doi: 10.1186/s44263-024-00049-2 (PMC11622986; doi:10.1186/s44263-024-00049-2)
Supplement: Supplementary file 1 — Additional file 1: Table S1. Participants responses in percentage reporting the van Rie stigma scale at baseline and follow-up. Table S2. Participants responses in percentage reporting for each Patient Health Questionnaire question at baseline and follow-up. Table S3. Participants responses in percentage reporting the experience of quality of life at baseline and follow-up. [file 44263_2024_49_MOESM1_ESM.docx]

**Stigma, depression and quality of life among people with pulmonary tuberculosis diagnosed through active and passive case finding in Nepal: a prospective cohort study**

**Additional file 1**

Table S1: Participants responses in percentage reporting the van Rie stigma scale at baseline and follow-up

|  |  |  | **All (n=221)** | | | | |  | **ACF (n=111)** | | | | | |  | | **PCF (n=110)** | | | | | |  |
| --- | --- | --- | --- | --- | --- | --- | --- | --- | --- | --- | --- | --- | --- | --- | --- | --- | --- | --- | --- | --- | --- | --- | --- |
|  |  |  | **Baseline** |  | **Follow-up** |  | **Change** |  | | **Baseline** |  | **Follow-up** |  | **Change** | |  | | **Baseline** |  | **Follow-up** |  | **Change** | |
|  |  |  | n (%) |  | n (%) |  | n (%) |  | | n (%) |  | n (%) |  | n (%) | |  | | n (%) |  | n (%) |  | n (%) | |
|  |  |  |  |  |  |  |  |  | |  |  |  |  |  | |  | |  |  |  |  |  | |
| Some people prefer not to have individuals with TB living in their community | Strongly agree |  | 17 (8) |  | 11 (5) |  | -6 (-3) |  | | 11(10) |  | 7 (6) |  | -4 (-4) | |  | | 6 (5) |  | 4 (3.5) |  | -2 (-1.5) | |
|  | Agree |  | 60 (28) |  | 71 (32) |  | 11 (4) |  | | 31(28) |  | 34 (31) |  | 3 (3) | |  | | 29 (26) |  | 37 (33.5) |  | 8 (6.5) | |
|  | Neither agree nor disagree |  | 2 (1) |  | 4 (2) |  | 2 (1) |  | | 0 (0) |  | 2 (2) |  | 2 (2) | |  | | 2 (2) |  | 2 (2) |  | 0 (0) | |
|  | Disagree |  | 58 (26) |  | 58 (26) |  | 0 (0) |  | | 23(21) |  | 24(21.5) |  | 1 (0.5) | |  | | 35 (32) |  | 34 (31) |  | -1 (-1) | |
|  | Strongly disagree |  | 84 (38) |  | 77 (35) |  | -7 (-3) |  | | 46(41) |  | 44(39.5) |  | -2 (-1.5) | |  | | 38 (35) |  | 33 (30) |  | -5 (-5) | |
|  | Missing response |  | 0 (0) |  | 0 (0) |  | 0 (0) |  | | 0 (0) |  | 0 (0) |  | 0 (0) | |  | | 0 (0) |  | 0 (0) |  | 0 (0) | |
|  |  |  |  |  |  |  |  |  | |  |  |  |  |  | |  | |  |  |  |  |  | |
| Some people keep their distance from individuals with TB disease | Strongly agree |  | 27 (12) |  | 28 (13) |  | 1 (1) |  | | 19 (17) |  | 12 (11) |  | -7 (-6) | |  | | 8 (7) |  | 16 (14.5) |  | 8 (7.5) | |
|  | Agree |  | 67 (30) |  | 60 (27) |  | -7 (-3) |  | | 30 (27) |  | 30 (27) |  | 0 (0) | |  | | 37 (34) |  | 30 (27) |  | -7 (-7) | |
|  | Neither agree nor disagree |  | 2 (1) |  | 2 (1) |  | 0 (0) |  | | 0 (0) |  | 0 (0) |  | 0 (0) | |  | | 2 (2) |  | 2 (2) |  | 0 (0) | |
|  | Disagree |  | 65 (29) |  | 78 (35) |  | 13 (6) |  | | 27 (24) |  | 40 (36) |  | 13 (12) | |  | | 38 (34) |  | 38 (34.5) |  | 0 (0) | |
|  | Strongly disagree |  | 60 (27) |  | 53 (24) |  | -7 (-3) |  | | 35 (32) |  | 29 (26) |  | -6 (-6) | |  | | 25 (23) |  | 24 (22) |  | -1 (-1) | |
|  | Missing response |  | 0 (0) |  | 0 (0) |  | 0 (0) |  | | 0 (0) |  | 0 (0) |  | 0 (0) | |  | | 0 (0) |  | 0 (0) |  | 0 (0) | |
|  |  |  |  |  |  |  |  |  | |  |  |  |  |  | |  | |  |  |  |  |  | |
| Some people feel uncomfortable when they are close to an individual with TB | Strongly agree |  | 28 (13) |  | 17 (8) |  | -11 (-5) |  | | 15 (13) |  | 11 (10) |  | -4 (-3) | |  | | 13 (12) |  | 6 (5.5) |  | -7 (-6.5) | |
|  | Agree |  | 67 (30) |  | 76 (34.5) |  | 9 (4.5) |  | | 32 (29) |  | 33 (30) |  | 1 (1) | |  | | 35 (32) |  | 43 (39) |  | 8 (7) | |
|  | Neither agree nor disagree |  | 2 (1) |  | 3 (1) |  | 1 (1) |  | | 0 (0) |  | 2 (2) |  | 2 (2) | |  | | 2 (2) |  | 1 (1) |  | -1 (-1) | |
|  | Disagree |  | 68 (31) |  | 71 (32) |  | 3 (1) |  | | 32 (29) |  | 31 (28) |  | -1 (-1) | |  | | 36 (33) |  | 40 (36) |  | 4 (4) | |
|  | Strongly disagree |  | 56 (25) |  | 54 (24) |  | -2 (-1) |  | | 32 (29) |  | 34 (31) |  | 2 (2) | |  | | 24 (22) |  | 20 (18) |  | -4 (-4) | |
|  | Missing |  | 0 (0) |  | 0 (0) |  | 0 (0) |  | | 0 (0) |  | 0 (0) |  | 0 (0) | |  | | 0 (0) |  | 0 (0) |  | 0 (0) | |
|  |  |  |  |  |  |  |  |  | |  |  |  |  |  | |  | |  |  |  |  |  | |
| Some people do not want to talk to individuals with TB | Strongly agree |  | 24 (11) |  | 21 (9.5) |  | -3 (1.5) |  | | 12 (11) |  | 13 (12) |  | 1 (1) | |  | | 12 (11) |  | 8 (7) |  | -4 (-4) | |
|  | Agree |  | 55 (25) |  | 53 (24) |  | -2 (-1) |  | | 25 (22.5) |  | 23 (21) |  | -2 (-1.5) | |  | | 30 (27) |  | 30 (27) |  | 0 (0) | |
|  | Neither agree nor disagree |  | 4 (2) |  | 4 (2) |  | 0 (0) |  | | 2 (2) |  | 2 (2) |  | 0 (0) | |  | | 2 (2) |  | 2 (2) |  | 0 (0) | |
|  | Disagree |  | 72 (32) |  | 82 (37) |  | 10 (5) |  | | 37 (33) |  | 38 (34) |  | 1 (1) | |  | | 35 (32) |  | 44 (40) |  | 9 (8) | |
|  | Strongly disagree |  | 66 (30) |  | 61 (27.5) |  | -5 (2.5) |  | | 35 (31.5) |  | 35 (31) |  | 0 (-0.5) | |  | | 31 (28) |  | 26 (24) |  | -5 (-4) | |
|  | Missing |  | 0 (0) |  | 0 (0) |  | 0 (0) |  | | 0 (0) |  | 0 (0) |  | 0 (0) | |  | | 0 (0) |  | 0 (0) |  | 0 (0) | |
|  |  |  |  |  |  |  |  |  | |  |  |  |  |  | |  | |  |  |  |  |  | |
| If a person has TB, some members of the community will behave differently in relation to that person for the rest of their life | Strongly agree |  | 15 (7) |  | 15 (6.5) |  | 0 (0) |  | | 7 (6) |  | 7 (6) |  | 0 (0) | |  | | 8 (7) |  | 8 (7) |  | 0 (0) | |
|  | Agree |  | 53 (24) |  | 44 (20) |  | -9 (-4) |  | | 24 (21.5) |  | 22 (20) |  | -2 (-1.5) | |  | | 29 (26) |  | 22 (20) |  | -7 (-6) | |
|  | Neither agree nor disagree |  | 3 (1) |  | 8 (3.5) |  | 5 (2.5) |  | | 3 (3) |  | 5 (5) |  | 2 (2) | |  | | 0 (0) |  | 3 (3) |  | 3 (3) | |
|  | Disagree |  | 77 (35) |  | 88 (40) |  | 11 (5) |  | | 34 (30.5) |  | 39 (35) |  | 5 (3.5) | |  | | 43 (39) |  | 49 (44.5) |  | 6 (5.5) | |
|  | Strongly disagree |  | 73 (33) |  | 66 (30) |  | 7 (3) |  | | 43 (39) |  | 38 (34) |  | -5 (-5) | |  | | 30 (27) |  | 28 (25.5) |  | -2 (-2) | |
|  | Missing |  | 0 (0) |  | 0 (0) |  | 0 (0) |  | | 0 (0) |  | 0 (0) |  | 0 (0) | |  | | 0 (0) |  | 0 (0) |  | 0 (0) | |
|  |  |  |  |  |  |  |  |  | |  |  |  |  |  | |  | |  |  |  |  |  | |
| Some people fear individuals who have TB | Strongly agree |  | 19 (9) |  | 16 (7) |  | -3 (-2) |  | | 13 (11.5) |  | 9 (8) |  | -4 (-3.5) | |  | | 6 (5.5) |  | 7 (6) |  | 1 (0.5) | |
|  | Agree |  | 73 (33) |  | 65 (29) |  | -8 (-4) |  | | 32 (29) |  | 34 (31) |  | 2 (2) | |  | | 41 (37) |  | 31 (28) |  | -10 (-9) | |
|  | Neither agree nor disagree |  | 5 (2) |  | 8 (4) |  | 3 (2) |  | | 2 (2) |  | 2 (2) |  | 0 (0) | |  | | 3 (3) |  | 6 (6) |  | 3 (3) | |
|  | Disagree |  | 68 (31) |  | 86 (39) |  | 18 (8) |  | | 30 (27) |  | 37 (33) |  | 7 (6) | |  | | 38 (34.5) |  | 49 (45) |  | 11 (10.5) | |
|  | Strongly disagree |  | 56 (25) |  | 46 (21) |  | -9 (-4) |  | | 34 (30.5) |  | 29 (26) |  | -5 (-4.5) | |  | | 22 (20) |  | 17 (15) |  | -5 (-4.5) | |
|  | Missing |  | 0 (0) |  | 0 (0) |  | 0 (0) |  | | 0 (0) |  | 0 (0) |  | 0 (0) | |  | | 0 (0) |  | 0 (0) |  | 0 (0) | |
|  |  |  |  |  |  |  |  |  | |  |  |  |  |  | |  | |  |  |  |  |  | |
| Some people with TB feel guilty about having TB | Strongly agree |  | 15 (7) |  | 22 (10) |  | 7 (3) |  | | 9 (8) |  | 11 (10) |  | 2 (2) | |  | | 6 (5) |  | 11 (10) |  | 5 (5) | |
|  | Agree |  | 95 (43) |  | 72 (33) |  | -13 (-10) |  | | 48 (43) |  | 39 (35) |  | -9 (-8) | |  | | 47 (43) |  | 33 (30) |  | -13 (-13) | |
|  | Neither agree nor disagree |  | 5 (2) |  | 11 (5) |  | 6 (3) |  | | 3 (3) |  | 3 (3) |  | 0 (0) | |  | | 2 (2) |  | 8 (7) |  | 6 (5) | |
|  | Disagree |  | 55 (25) |  | 71 (32) |  | 16 (7) |  | | 19 (17) |  | 32 (29) |  | 12 (12) | |  | | 36 (33) |  | 39 (35) |  | 3 (2) | |
|  | Strongly disagree |  | 51 (23) |  | 45 (20) |  | -6 (-3) |  | | 32 (29) |  | 26 (23) |  | 6 (6) | |  | | 19 (17) |  | 19 (17) |  | 0 (0) | |
|  | Missing |  | 0 (0) |  | 0 (0) |  | 0 (0) |  | | 0 (0) |  | 0 (0) |  | 0 (0) | |  | | 0 (0) |  | 0 (0) |  | 0 (0) | |
|  |  |  |  |  |  |  |  |  | |  |  |  |  |  | |  | |  |  |  |  |  | |
| Some people with TB feel hurt with the way other people react when they learnt that they have TB | Strongly agree |  | 19 (9) |  | 14 (6) |  | -5 (-3) |  | | 12 (11) |  | 10 (9) |  | -2 (-2) | |  | | 7 (6) |  | 4 (4) |  | -2 (-2) | |
|  | Agree |  | 65 (29.5) |  | 72 (33) |  | 7 (3.5) |  | | 32 (29) |  | 31 (28) |  | -1 (-1) | |  | | 33 (30) |  | 41 (37) |  | 8 (7) | |
|  | Neither agree nor disagree |  | 7 (3) |  | 12 (5) |  | 5 (2) |  | | 3 (3) |  | 4 (4) |  | 1 (1) | |  | | 4 (4) |  | 8 (7) |  | 4 (4) | |
|  | Disagree |  | 72 (32.5) |  | 79 (36) |  | 7 (3.5) |  | | 28 (25) |  | 40 (36) |  | 12 (11) | |  | | 44 (40) |  | 39 (35.5) |  | -5 (-4.5) | |
|  | Strongly disagree |  | 58 (26) |  | 44 (20) |  | -14 (-6) |  | | 36 (32) |  | 26 (23) |  | -10 (-9) | |  | | 22 (20) |  | 18 (16.5) |  | -4 (-3.5) | |
|  | Missing |  | 0 (0) |  | 0 (0) |  | 0 (0) |  | | 0 (0) |  | 0 (0) |  | 0 (0) | |  | | 0 (0) |  | 0 (0) |  | 0 (0) | |
|  |  |  |  |  |  |  |  |  | |  |  |  |  |  | |  | |  |  |  |  |  | |
| Some people with TB fear telling people outside of their household that they have TB disease | Strongly agree |  | 16 (7) |  | 15 (7) |  | -1 (0) |  | | 11 (10) |  | 9 (8) |  | -2 (-2) | |  | | 5 (4.5) |  | 6 (5) |  | 1 (0.5) | |
|  | Agree |  | 98 (44) |  | 85 (38.5) |  | -13 (-4.5) |  | | 45 (40.5) |  | 38 (34) |  | -7 (-6.5) | |  | | 53 (48) |  | 47 (43) |  | -6 (-5) | |
|  | Neither agree nor disagree |  | 2 (1) |  | 2 (1) |  | 0 (0) |  | | 2 (2) |  | 0 (0) |  | -2 (-2) | |  | | 0 (0) |  | 2 (2) |  | 2 (2) | |
|  | Disagree |  | 59 (27) |  | 78 (35) |  | 19 (8) |  | | 24 (21.5) |  | 39 (35) |  | 15 (13.5) | |  | | 35 (32) |  | 39 (35.5) |  | 4 (3.5) | |
|  | Strongly disagree |  | 46 (21) |  | 41 (18.5) |  | -5 (2.5) |  | | 29 (26) |  | 25 (23) |  | -4 (-3) | |  | | 17 (15.5) |  | 16 (14.5) |  | -1 (-1) | |
|  | Missing |  | 0 (0) |  | 0 (0) |  | 0 (0) |  | | 0 (0) |  | 0 (0) |  | 0 (0) | |  | | 0 (0) |  | 0 (0) |  | 0 (0) | |
|  |  |  |  |  |  |  |  |  | |  |  |  |  |  | |  | |  |  |  |  |  | |
| Some people with TB fear telling their household that they have TB disease | Strongly agree |  | 10 (4.5) |  | 3 (1) |  | -7 (-3.5) |  | | 7 (6) |  | 3 (3) |  | -4 (-3) | |  | | 3 (3) |  | 0 (0) |  | -3 (-3) | |
|  | Agree |  | 37 (16.5) |  | 33 (15) |  | -4 (-1.5) |  | | 21 (19) |  | 13 (12) |  | -8 (-7) | |  | | 16 (14) |  | 20 (18) |  | 4 (4) | |
|  | Neither agree nor disagree |  | 4 (2) |  | 3 (1) |  | -1 (-1) |  | | 1 (1) |  | 2 (2) |  | 1 (1) | |  | | 3 (3) |  | 1 (1) |  | -2 (-2) | |
|  | Disagree |  | 88 (40) |  | 117 (53) |  | 29 (13) |  | | 38 (34) |  | 64 (58) |  | 26 (24) | |  | | 50 (45.5) |  | 53 (48) |  | 3 (2.5) | |
|  | Strongly disagree |  | 82 (37) |  | 65 (29) |  | -17 (-12) |  | | 44 (40) |  | 29 (26) |  | -15 (14) | |  | | 38 (34.5) |  | 36 (33) |  | -2 (-1.5) | |
|  | Missing |  | 0 (0) |  | 0 (0) |  | 0 (0) |  | | 0 (0) |  | 0 (0) |  | 0 (0) | |  | | 0 (0) |  | 0 (0) |  | 0 (0) | |
|  |  |  |  |  |  |  |  |  | |  |  |  |  |  | |  | |  |  |  |  |  | |

Table S2: Participants responses in percentage reporting for each Patient Health Questionnaire question at baseline and follow-up

| **Characteristics** | **Baseline** | | | | | |  | **Follow up** | | | | |
| --- | --- | --- | --- | --- | --- | --- | --- | --- | --- | --- | --- | --- |
|  |  | **ACF**  **(n=11)** |  | **PCF (n=110)** |  | **Total**  **(n=221)** |  | **ACF**  **(n=11)** |  | **PCF (n=110)** |  | **Total**  **(n=221)** |
| Experience of heart mind problem in past two weeks |  |  |  |  |  |  |  |  |  |  |  |  |
|  | Yes | 38(34) |  | 32 (29) |  | 70 (32) |  | 26(23) |  | 38 (35) |  | 64 (29) |
|  | No | 73(66) |  | 78 (71) |  | 151 (68) |  | 85(77) |  | 72 (65) |  | 157 (71) |
| Not happy in past two weeks |  |  |  |  |  |  |  |  |  |  |  |  |
|  | Not at all | 19(17) |  | 12 (11) |  | 31 (14) |  | 13(12) |  | 16 (15) |  | 29 (13) |
|  | Several days | 9 (8) |  | 15 (14) |  | 24 (11) |  | 11 (10) |  | 17 (16) |  | 28 (13) |
|  | More than half the days | 7 (6) |  | 3 (3) |  | 10 (5) |  | 0 (0) |  | 2 (2) |  | 2 (1) |
|  | Nearly every day | 3 (3) |  | 2 (2) |  | 5 (2) |  | 1 (1) |  | 0 (0) |  | 1 (1) |
|  | Missing | 0 (0) |  | 0 (0) |  | 0 (0) |  | 1 (1) |  | 3 (3) |  | 4 (2) |
| Felt frustrated, despairing or incapable of doing anything? | | |  |  |  |  |  |  |  |  |  |  |
|  | Not at all | 17(15) |  | 16 (15) |  | 33 (15) |  | 11(10) |  | 23 (21) |  | 34 (15) |
|  | Several days | 13(12) |  | 12 (11) |  | 25 (11) |  | 11(10) |  | 8 (7) |  | 19 (9) |
|  | More than half the days | 5 (5) |  | 3 (3) |  | 8 (4) |  | 3 (3) |  | 4 (4) |  | 7 (3) |
|  | Nearly every day | 3 (3) |  | 0 (0) |  | 3 (1) |  | 0 (0) |  | 0 (0) |  | 0 (0) |
|  | Missing | 0 (0) |  | 1 (1) |  | 1 (1) |  | 1 (1) |  | 3 (3) |  | 4 (2) |
| Problems with sleep n(%) |  |  |  |  |  |  |  |  |  |  |  |  |
|  | Not at all | 21(19) |  | 13 (12) |  | 34 (15) |  | 8 (7) |  | 19 (17) |  | 27 (12) |
|  | Several days | 8 (7) |  | 13 (12) |  | 21 (10) |  | 11(10) |  | 12 (11) |  | 23 (10) |
|  | More than half the days | 6 (5) |  | 3 (3) |  | 9 (4) |  | 4 (4) |  | 4 (4) |  | 8 (4) |
|  | Nearly every day | 3 (3) |  | 3 (3) |  | 6 (3) |  | 2 (2) |  | 0 (0) |  | 2 (1) |
|  | Missing | 0 (0) |  | 0 (0) |  | 0 (0) |  | 1 (1) |  | 3 (3) |  | 4 (2) |
| Lack of energy |  |  |  |  |  |  |  |  |  |  |  |  |
|  | Not at all | 16(14) |  | 12 (11) |  | 28 (13) |  | 9 (8) |  | 18 (16) |  | 27 (12) |
|  | Several days | 12(11) |  | 11 (10) |  | 23 (10) |  | 13(12) |  | 11 (10) |  | 24 (11) |
|  | More than half the days | 7 (6) |  | 8 (7) |  | 15 (7) |  | 3 (3) |  | 5 (5) |  | 8 (4) |
|  | Nearly every day | 3 (3) |  | 1 (1) |  | 4 (2) |  | 0 (0) |  | 1 (1) |  | 1 (1) |
|  | Missing | 0 (0) |  | 0 (0) |  | 0 (0) |  | 1 (1) |  | 3 (3) |  | 4 (2) |
| Lack of appetite |  |  |  |  |  |  |  |  |  |  |  |  |
|  | Not at all | 17(15) |  | 10 (9) |  | 27 (12) |  | 10 (9) |  | 14 (13) |  | 24 (11) |
|  | Several days | 5 (5) |  | 12 (11) |  | 17 (8) |  | 7 (6) |  | 15 (14) |  | 22 (10) |
|  | More than half the days | 8 (7) |  | 6 (6) |  | 14 (6) |  | 3 (3) |  | 2 (2) |  | 5 (2) |
|  | Nearly every day | 7 (6) |  | 4 (4) |  | 11 (5) |  | 5 (5) |  | 4 (4) |  | 9 (4) |
|  | Missing | 1 (1) |  | 0 (0) |  | 1 (1) |  | 1 (1) |  | 3 (3) |  | 4 (2) |
| Difficulty to focus or concentrate | |  |  |  |  |  |  |  |  |  |  |  |
|  | Not at all | 27(24) |  | 18 (16) |  | 45 (20) |  | 16(14) |  | 21 (19) |  | 37 (17) |
|  | Several days | 5 (5) |  | 12 (11) |  | 17 (8) |  | 7 (6) |  | 12 (11) |  | 19 (9) |
|  | More than half the days | 5 (5) |  | 2 (2) |  | 7 (3) |  | 2 (2) |  | 2 (2) |  | 4 (2) |
|  | Nearly every day | 1 (1) |  | 0 (0) |  | 1 (1) |  | 0 (0) |  | 0 (0) |  | 0 (0) |
|  | Missing | 0 (0) |  | 0 (0) |  | 0 (0) |  | 1 (1) |  | 3 (3) |  | 4 (2) |
| Talking softly, walking slowly |  |  |  |  |  |  |  |  |  |  |  |  |
|  | Not at all | 33(30) |  | 22 (20) |  | 55 (25) |  | 23(21) |  | 27 (25) |  | 50 (23) |
|  | Several days | 2 (2) |  | 10 (9) |  | 12 (5) |  | 1 (1) |  | 7 (6) |  | 8 (4) |
|  | More than half the days | 2 (2) |  | 0 (0) |  | 2 (1) |  | 1 (1) |  | 1 (1) |  | 2 (1) |
|  | Nearly every day | 1 (1) |  | 0 (0) |  | 1 (1) |  | 0 (0) |  | 0 (0) |  | 0 (0) |
|  | Missing | 0 (0) |  | 0 (0) |  | 0 (0) |  | 1 (1) |  | 3 (3) |  | 4 (2) |
| Feeling of hurting oneself |  |  |  |  |  |  |  |  |  |  |  |  |
|  | Not at all | 34(31) |  | 29 (26) |  | 63 (29) |  | 23(21) |  | 34 (31) |  | 57 (26) |
|  | Several days | 2 (2) |  | 3 (3) |  | 5 (2) |  | 1 (1) |  | 1 (1) |  | 2 (1) |
|  | More than half the days | 2 (2) |  | 0 (0) |  | 2 (1) |  | 1 (1) |  | 0 (0) |  | 1 (1) |
|  | Nearly every day | 0 (0) |  | 0 (0) |  | 0 (0) |  | 0 (0) |  | 0 (0) |  | 1 (1) |
|  | Missing | 0 (0) |  | 0 (0) |  | 0 (0) |  | 1 (1) |  | 3 (3) |  | 4 (2) |

Table S3: Participants responses in percentage reporting the experience of quality of life at baseline and follow-up

|  |  | **Baseline** | | | |  | **Follow up** | | | |
| --- | --- | --- | --- | --- | --- | --- | --- | --- | --- | --- |
| Best describe walking today |  | **ACF** | **PCF** | **Total** | **p-value** |  | **ACF** | **PCF** | **Total** | **p-value** |
|  | Unable | 1 (1) | 1 (1) | 2 (1) | 0.84 |  | 0 | 0 | 0 | 0.25 |
|  | Severe problems | 3 (3) | 1 (1) | 4 (2) |  |  | 2 (2) | 0 | 2 (1) |  |
|  | Moderate problems | 2 (2) | 1 (1) | 3 (1) |  |  | 2 (2) | 0 | 2 (1) |  |
|  | Slight problems | 18 (16) | 20 (18) | 38 (17) |  |  | 12 (11) | 11 (10) | 23 (10) |  |
|  | No problem | 87 (78) | 87 (79) | 174 (79) |  |  | 95 (86) | 99 (90) | 194 (88) |  |
| Best describe self-care today |  |  |  |  |  |  |  |  |  |  |
|  | Unable | 2 (2) | 0 | 2 (1) | 0.34 |  | 0 | 0 | 0 | 0.064 |
|  | Severe problems | 1 (1) | 0 (0) | 1 (0.5) |  |  | 0 | 0 | 0 |  |
|  | Moderate problems | 1 (1) | 0 (0) | 1 (0.5) |  |  | 3 (3) | 0 | 3 (1) |  |
|  | Slight problems | 16 (14) | 13 (12) | 29 (13) |  |  | 8 (7) | 3 (3) | 11 (5) |  |
|  | No problem | 91 (82) | 97 (88) | 188 (85) |  |  | 100 (90) | 107 (97) | 207  (94) |  |
| Best describe usual activities today |  |  |  |  |  |  |  |  |  |  |
|  | Unable | 1 (1) | 0 | 1 (0.5) | 0.15 |  | 1 (1) | 0 | 1 (0.5) | 0.51 |
|  | Severe problems | 3 (3) | 0 | 3 (1) |  |  | 0 | 0 | 0 |  |
|  | Moderate problems | 3 (3) | 1 (1) | 4 (2) |  |  | 1 (1) | 0 | 1 (0.5) |  |
|  | Slight problems | 20 (18) | 14 (13) | 34 (15) |  |  | 8 (7) | 6 (5) | 14 (6) |  |
|  | No problem | 84 (76) | 95 (86) | 179 (81) |  |  | 101 (91) | 104 (94) | 205 (93) |  |
| Best describe pain discomfort today |  |  |  |  |  |  |  |  |  |  |
|  | Unable | 1 (1) | 1 (1) | 2 (1) | 0.79 |  | 0 | 0 | 0 | 0.66 |
|  | Severe problems | 1 (1) | 2 (2) | 3 (1) |  |  | 1 (1) | 0 | 1 (0.5) |  |
|  | Moderate problems | 5 (4) | 4 (4) | 9 (4) |  |  | 1 (1) | 1 (1) | 2 (1) |  |
|  | Slight problems | 27 (24) | 20 (18) | 47 (21) |  |  | 17 (15) | 13 (12) | 30 (14) |  |
|  | No problem | 77 (69) | 83 (75) | 160 (72) |  |  | 92 (83) | 96 (87) | 188 (85) |  |
| Best describe anxiety depression today |  |  |  |  |  |  |  |  |  |  |
|  | Unable | 1 (1) | 1 (1) | 2 (1) | 0.70 |  | 0 | 1 (1) | 1 (0.5) | 0.367 |
|  | Severe problems | 5 (4) | 2 (2) | 7 (3) |  |  | 0 | 0 | 0 |  |
|  | Moderate problems | 2 (2) | 4 (4) | 6 (3) |  |  | 0 | 2 (2) | 2 (1) |  |
|  | Slight problems | 22 (20) | 25 (23) | 47 (21) |  |  | 15 (13) | 16 (15) | 31 (14) |  |
|  | No problem | 81 (73) | 78 (71) | 159 (72) |  |  | 96 (86) | 91 (83) | 187 (85) |  |
