# Supplementary material for: Stigma, depression, and quality of life among people with pulmonary tuberculosis diagnosed through active and passive case finding in Nepal: a prospective cohort study
Source: BMC Glob Public Health. 2024 Mar 24;2:20. doi: 10.1186/s44263-024-00049-2 (PMC11622986; doi:10.1186/s44263-024-00049-2)
Supplement: Supplementary file 2 — Additional file 2: Figure S1. Correlation between baseline depression and stigma scores amongst participants who screened positive for depression (n=71). [file 44263_2024_49_MOESM2_ESM.docx]

**Stigma, depression and quality of life among people with pulmonary tuberculosis diagnosed through active and passive case finding in Nepal: a prospective cohort study**

**Additional file 2**

Fig S1: Correlation between baseline depression and stigma scores amongst participants who screened positive for depression (n=71)


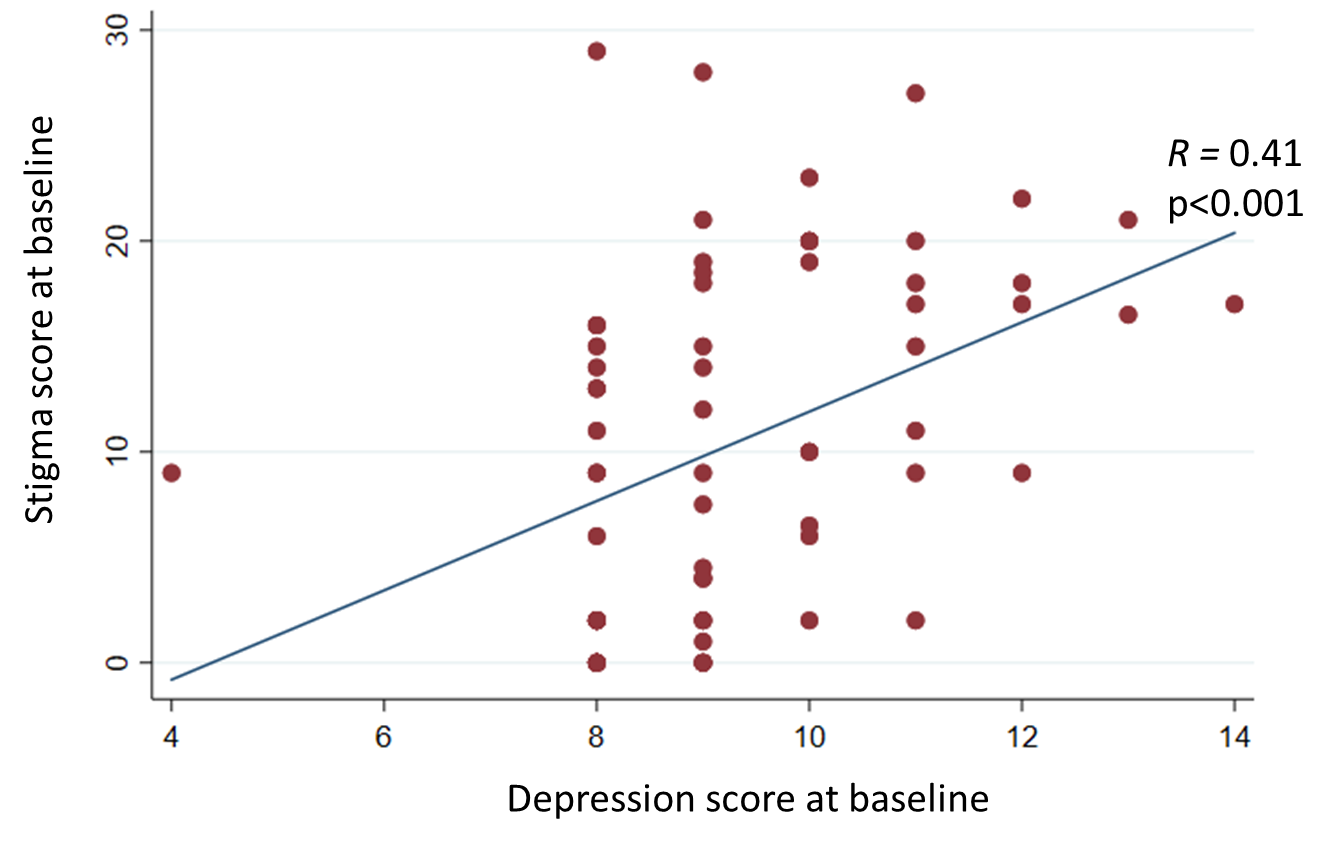


*Legend: The figure shows the Spearman’s coefficient (r) with p value corrected by Bonferroni adjustment of the correlation of stigma score measured by adapted Van Rie Stigma Scale with depression measured by PHQ-9*
